# Maximizing Energy Content and CO_2_ Bio-fixation Efficiency of an Indigenous Isolated Microalga *Parachlorella kessleri* HY-6 Through Nutrient Optimization and Water Recycling During Cultivation

**DOI:** 10.3389/fbioe.2021.804608

**Published:** 2022-02-10

**Authors:** Wasif Farooq

**Affiliations:** ^1^ Chemical Engineering Department, King Fahd University of Petroleum and Minerals (KFUPM), Dhahran, Saudi Arabia; ^2^ Integrated Research Centre for Membranes and Water Security (IRC-MWS ), King Fahd University of Petroleum and Minerals (KFUPM), Dhahran, Saudi Arabia

**Keywords:** microalgae, CO_2_ bio-fixation, light intensity, energy content, biofuel production

## Abstract

An alternative source of energy and materials with low negative environmental impacts is essential for a sustainable future. Microalgae is a promising candidate in this aspect. The focus of this study is to optimize the supply of nitrogen and carbon dioxide during the cultivation of locally isolated strain *Parachlorella kessleri* HY-6. This study focuses on optimizing nitrogen and CO_2_ supply based on total biomass and biomass per unit amount of nitrogen and CO_2_. Total biomass increased from 1.23 to 2.30 g/L with an increase in nitrogen concentration from 15.8 to 47.4 mg/L. However, biomass per unit amount of nitrogen supplied was higher at low nitrogen content. Biomass and CO_2_ fixation rate increased at higher CO_2_ concentrations in bubbling air, but CO_2_ fixation efficiency decreased drastically. Finally, the energy content of biomass increased with increases in both nitrogen and CO_2_ supply. This work thoroughly analyzed the biomass composition *via* ultimate, proximate, and biochemical analysis. Water is recycled three times for cultivation at three different nitrogen levels. Microalgae biomass increased during the second recycling and then decreased drastically during the third. Activated carbon helped remove the organics after the third recycling to improve the water recyclability. This study highlights the importance of selecting appropriate variables for optimization by considering net energy investment in terms of nutrients (as nitrogen) and CO_2_ fixation efficiency and effective water recycling.

## Introduction

Greenhouse gas emissions are increasing, and the CO_2_ concentration has reached 410 ppm and is expected to reach 450 ppm by 2035 (Bernard 2011). The highest contributors to CO_2_ emission are the power generation, industrial, and transportation sectors. Carbon capture and utilization (CCU) is an emerging approach to mitigate the effect of anthropogenic CO_2_ emissions. CCU relies on CO_2_ capture from a point source emitter and converts it to valuable and innovative new products through chemical or biological routes (Chen L. et al., 2017). CO_2_ is captured and managed *via* pre-combustion, post-combustion, or oxyfuel combustion methods (Coimbra et al., 2019). Several options are proposed for CCU to convert CO_2_ to various valuable molecules, such as bio-fixation of CO_2_ or direct catalytic conversion. Biological systems are more competent in utilizing CO_2_ than chemical conversion techniques. Different natural processes, such as forestation and ocean fertilization, and microbes or microalgae fixed and used CO_2_ (Daneshvar et al., 2022).

Microalgae are photosynthetic microorganisms that utilize CO_2_ as the primary carbon source, and their rate of fixing CO_2_ is 100 times faster than that of terrestrial plants. Most of the eukaryotic algae comprise of pyrenoids, a proteinaceous sub-cellular compartmentalized structure capable of fixing nearly 30–40% of atmospheric CO_2_ due to the presence of the Rubisco enzyme. The photosynthetic efficiency of microalgae is 10 times higher than that of terrestrial plants due to their energy-conserving structures and simple cell structure. Moreover, many microalgae did not need fresh water and fertilized land to preliterate and offer a rapid multiplication rate and arial productivity than higher plants (Singh and Ahluwalia, 2013). Microalgae can utilize CO_2_ as inorganic carbon in three modes: passive diffusion of CO_2_, active uptake of HCO_3_^−^, and CO_2_ or external carbonic anhydrase enzyme to the plasma membrane to facilitate HCO_3_^−^ (Colman et al., 2002). Many researchers have investigated microalgae as an alternative approach for CO_2_ capture and utilization. A recent detailed review on biobased CCU highlights the advantages and, most notably, the gaps in research and potential future research directions to improve the efficiency of the process, especially by integrating new innovative CO_2_ capture technologies with microalgae cultivation (Daneshvar et al., 2022).

Microalgae convert CO_2_ into several commercially valuable molecules, such as lipids, carbohydrates, proteins, and pigments, through photosynthesis (Chen L. et al., 2017). Moreover, first- and second-generation biofuel feedstocks are limited and unsustainable for long-term and high yield. Alternatively, third-generation biofuel feedstocks, such as algal biomass, are excellent alternative sources for biofuel production. Microalgae offer higher productivity, faster growth rates, and high photosynthetic efficiency than traditional crops. Microalgae can grow over a wide range of CO_2_ concentrations (1–40%) (Li et al., 2013). In addition, algae can be used to remove phosphorus, nitrogen, and heavy metals from wastewater (López Barreiro et al., 2015). Microalgae can grow on wastewater and under extreme environmental conditions (Farooq et al., 2013; Pires et al., 2014). Microalgae are cultivated mainly under three different modes: heterotrophic, photoautotrophic, and mixotrophic (Khalili et al., 2015). Expensive organic substrate and strict operational control are required to avoid undesirable bacterial contamination during mixotrophic and heterotrophic cultivation (Moon et al., 2014). The use of mixotrophic and heterotrophic cultivation modes will lose the advantage of CO_2_ fixation. A photoautotrophic method is preferred where natural light is the energy source, and CO_2_ is fixed during cultivation. Researchers are studying several parameters affecting the microalgal growth, biomass, and lipid production linked with CO_2_ capture. However, the commercialization of microalgae is still facing many challenges despite its favorable characteristics of CO_2_ capture and higher yield of biomass and biofuel (Mustapha et al., 2021).

The phototrophic growth of microalgae needs a substantial amount of nitrogen (and phosphorus-based fertilizer) and water. Various life cycle analysis studies reported very high water demand (Farooq et al., 2015a). Without adequate water reuse during cultivation, microalgae require approximately 3,000 L of water per liter of biodiesel. Therefore, water reuse is essential, and estimates showed that water reuse could reduce the consumption to <1,000 L of water per liter of biodiesel (Farooq et al., 2015b). Higher water demand puts stress on the water resources and will offset the benefits of CO_2_ fixation due to the energy required for acquiring water. Therefore, to maximize the CO_2_ fixation potential of microalgae, direct and indirect emissions of CO_2_ must be reduced. Synthetic fertilizers are produced by fossil fuel sources and result in CO_2_ emissions. Estimates showed that, for 1 kg of nitrogenous fertilizer, 49.9–63.2 MJ of fossil fuel energy is required. As a result, 3.39–6.92 kg of CO_2_ equivalent is emitted per kilogram of nitrogenous fertilizer (Brentrup et al., 2016). Though CO_2_ fixation rate by microalgae is reported widely in literature, limited data is available on factors affecting CO_2_ fixation efficiency (Leflay et al., 2021). Therefore, optimizing nutrient requirements and using unused nutrients in water through water recycling is inevitable. Thus, water reuse during cultivation is essential to minimize the energy to acquire water and reduce the stress on water bodies (Hadj-Romdhane et al., 2013). The microalgal photosynthetic mechanism is supported by a light-harvesting system containing various pigments. Variation in pigment content changes when microalgae are under stress. Chlorophyll is essential for effective photosynthesis and CO_2_ biological fixation (Chen B. et al., 2017).

Majorities of studies focused on nutrient optimization for biomass production without realizing their net impact on CO_2_ fixation efficiency and recyclability of water during cultivation. Notably, this study investigated the effect of four different nutrient regimes of nitrogen and phosphorus on overall energy contents as higher heating value (HHV) of biomass and CO_2_ fixation efficiency under six different CO_2_ concentrations at three different light intensities along with changes in water chemistry during growth for its potential reuse in the cultivation stage and subsequent CO_2_ fixation. More specifically, the impact of nutrients is investigated on variation in photosynthetic pigments, CO_2_ fixation rate, CO_2_ capture efficiency, and variation in water chemistry after growth under different light intensities.

## Materials and Methods

### Microalgae Growth

Locally isolated microalgae strain identified as *Parachlorella kessleri* HY-6 was cultivated using modified Bold’s basal (BBM) medium as described (Wadood et al., 2020). The BBM growth medium contains macronutrients (in mM) such as 3.38 NaNO_3_, 0.17 CaCl_2_·2H_2_O, 0.30 MgSO_4_·7H_2_O, 0.05 KH_2_PO_4_, 0.05 K_2_HPO_4_, 0.43 NaCl, 0.18 H_3_BO_3_, and EDTA solution (in mM) which contained 0.17 Na_2_EDTA·2H_2_O, 0.55 KOH, and ferric solution (in µM) which contained 17.9 FeSO_4_·7H_2_O. Essential micronutrients were supplied as trace metal element solution (in µM) containing 7.28 MnCl_2_·4H_2_O, 30.70 ZnSO_4_·7H_2_O, 1.68 Co(NO_3_)_2_·6H_2_O, and 6.29 CuSO_4_·5H_2_O. Microalgae were grown in 500-ml autoclaved Erlenmeyer flasks with a working volume of 350 ml at 25 ± 1°C under varying light intensities of 30, 60, and 100 μmol m^−2^ s^−1^. The culture was aerated at an average flow rate of 0.5 vvm with different percentages of CO_2_ (0–15%). Microalgae biomass was harvested at the end of a 12-day cultivation cycle using a centrifuge at 7,000 rpm for 4 min, followed by rinsing with distilled water to remove any residual salt. Biomass was dried at 105°C. Experiments on the effects of different total nitrogen (TN) concentrations were conducted with four different initial TN concentrations of 15.8, 31.6, 47.4, and 63.2 ppm. Microalgae growth was conducted under different ratios of nitrogen to phosphorus (N/P), such as 1:1, 5:1, 10:1, and 15:1, where total phosphorus (TP) was 47.4, 9.48, 4.74, and 3.16 ppm at a total nitrogen concentration of 47.4 ppm. The growth behavior of the microalgae at various CO_2_ amounts was tested by varying concentrations of CO_2_ (0, 2, 4, 6, 10, and 15%) diluted with air under initial TN and TP of 47.4 and 3.16 ppm, respectively.

### Determination of Biomass and Lipid Productivity

The growth of *P. kessleri* HY-6 was measured by optical density (OD) at 680 nm using a UV–vis spectrophotometer (Thermo Fisher Scientific Evolution 200 Series). The weight of dried biomass collected by centrifugation and dried at 105°C was measured for dry biomass calculation to establish the relationship between biomass and optical density. The relationship between the biomass concentration and OD at *A*_680__nm_ was found by appropriate standard calibration as shown in the following equation:

$${\text{OD}}_{680}=3.426\text{DW}\left(R^{2}=0.983\right)$$ (1)

DW is dry biomass in (g L^−1^). Lipid productivity was calculated by lipid content (%) multiplied by biomass productivity. Microalgae biomass productivity (mg L^−1^ day^−1^) was calculated by dividing the biomass per unit volume with time for cultivation as follows:

$$\text{P}=\frac{\Delta X}{\Delta \text{t}}$$ (2)

where ∆*X* is the biomass concentration (in mg L^−1^) within a cultivation period of ∆*t* (in days).

### Determination of Nitrogen and Phosphorus Removal Efficiency

TN and TP were analyzed every 48 h with a Hach analysis kit by using a DR3900 spectrophotometer. The culture cells were centrifuged at 4,500 rpm for 5 min and vacuum-filtered through a membrane filter (Whatman GF/F, 47 mm, nominal pore size: 0.7 μm). The filtered supernatant was used for the determination of TN and TP. The nitrogen and phosphorus removal efficiency was calculated using Eq. 3, where *C*_0_ and *C* are the nutrient values at *t*_0_ and *t*, respectively.

$$R_{\varepsilon }\left[\text{\%}\right]=\frac{\left|C_{0}-C\right|}{C_{0}}\times 100$$ (3)

### Determination of Pigment Content

Pigment content was measured using a UV/vis spectrophotometer (Thermo Fisher Scientific Evolution 200 Series). Pigments were extracted from dry cells using methanol (99.8%) with ultrasonication for 30 min and 45°C followed by centrifugation at 4,500 rpm for 3 min. Absorbance was corrected from turbidity by subtracting absorbencies at 750 nm. The absorption spectrum was collected in the range 400–750 nm where chlorophyll-a, chlorophyll-b, and photoprotective carotenoid concentrations were determined according to the following equations (19):

$$\text{Chlorophyll}\;\text{a}\left[\frac{\text{μg}}{\text{mL}}\right]=-8.0962\times A_{652}+16.5169\times A_{665}$$ (4)

$$\text{Chlorophyll}\;\text{b}\left[\frac{\text{μg}}{\text{mL}}\right]=27.4405\times A_{652}-12.1688\times A_{665}$$ (5)

$$\text{Carotenoids}\left[\frac{\text{μg}}{\text{mL}}\right]=4.0\times A_{480}$$ (6)

### Determination of CO_2_ Fixation Rate

The carbon content of the biomass was measured using an elemental analyzer (PerkinElmer 2400 Series II CHNS/O Elemental Analyzer, Perkin Elmer Corporation). Dried biomass samples were weighted up to 0.8–2.0 mg in pre-weighted and pre-cleaned tin capsules (5 × 8 mm, Perkin Elmer). The samples were then combusted at 1,000°C using pure helium as the carrier gas and pure oxygen as the combustion gas. The instrument was calibrated with acetanilide standards with delta-calibrated criteria of ±0.15 for carbon, ±3.75 for hydrogen, and ±0.16 for nitrogen. The rate of CO_2_ fixation (mg/L/day) and CO_2_ fixation efficiency is estimated by the following equation (20):

$${\text{R}}_{CO_{2}}=\text{P}\times {\text{C}}_{Carbon}\times \frac{M_{CO_{2}}}{{\text{M}}_{C}}\;$$ (7)

$${\text{CO}}_{2}\;\text{removal}\;\text{efficiency}\;\left(\text{\%}\right)\;=\;\frac{Total\;CO2\;biofixed\left(g\right)}{Total\;CO2\;input\left(g\right)}\times 100$$ (8)

where *P*, *C*_carbon_, *M*_CO2,_ and *M*_C_ are the biomass productivity, carbon content, molar mass of CO_2_, and carbon, respectively.

### Determination of Total Lipid and FAME Content

The total lipids were extracted from 50 mg of biomass in glass vials and quantified gravimetrically by the Bligh and Dyer method using 3.0 ml of (2:1, v/v) chloroform–methanol solution in two cycles. Biomass was sonicated in an ultra-sonicator to enhance lipid extraction at 50°C for 30 min. The extracted lipids in glass vials were dried using a vacuum oven at 95°C, and later the glass vials were weighted to calculate the lipid content as follows:

$$\text{Lipid}\;\text{Content}=\frac{\left({\text{m}}_{\text{Extracted}\;\text{Lipid}}-{\text{m}}_{\text{Glass}\;\text{Vial}}\right)}{{\text{m}}_{\text{Dry}\;\text{Biomass}}}$$ (9)

Fatty acid methyl esters (FAME) were converted to biodiesel by transesterification of dried microalgal biomass with sulfuric acid. Briefly, 26 mg of dry biomass was directly trans-esterified with 0.3 ml of H_2_SO_4_ with 3.0 ml of 9:1 methanol-dimethyl sulfoxide mixture at 60°C. After that, 1.0 ml of the trans-esterified mixture was filtered with a 0.2-µm syringe filter (Whatman, Springfield Mill, United Kingdom). The samples were centrifuged at 4,500 rpm for 3 min for phase separation. The upper organic layer was collected in a new vial, and then the FAME in the organic phase was analyzed by gas chromatography–mass spectrometry (GC-6890N, MSD-5975B, Agilent Technologies, United States). Each FAME was identified and quantified by comparing the respective peak area and retention time with the standard. The FAME percentage was calculated as per the following equation (Salam et al., 2016):

$$\text{FAME}\left[\text{\%}\right]=\frac{{\text{Area}}_{\text{FAME}}}{{\text{Area}}_{\text{FM}}}\times \frac{{\text{C}}_{\text{FM}}\times {\text{V}}_{\text{FM}}}{{\text{m}}_{\text{Dry}\;\text{Biomass}}}\times 100$$ (10)

where Area_FAME_, Area_FM_, *C*_FM_, *V*_FM_, and *m*_Dry biomass_ are the total peak area, peak area corresponding to the pure FAME mix solution, concentration of the pure FAME mix solution (in mg/ml), volume of the pure FAME mix solution (in ml), and the mass of the dried microalgal sample (in mg), respectively.

### Total Protein Analysis

Protein content was estimated using the recommended Kjeldahl conversion factor of 5.95 for elemental nitrogen to protein estimation (López et al., 2010). Total nitrogen of biomass was measured by an elemental analyzer (PerkinElmer 2400 Series II CHNS/O Elemental Analyzer, Perkin Elmer Corporation).

### Proximate and Ultimate Analysis of Biomass

Thermogravimetric analysis using TA Instruments SDT 600 measured the fixed carbon, moisture, ash content, and volatile matter of biomass. Moisture content and volatile matter were based on when the weight change was almost zero at 100 and 850°C, respectively. Post-pyrolysis combustion proceeded under air to 800°C, followed by a stepwise function to 850°C to estimate the fixed carbon and ash content. The elemental analyzer determined total carbon, hydrogen, and nitrogen (PerkinElmer 2400 Series II CHNS/O Elemental Analyzer, Perkin Elmer Corporation).

### Analysis of Water for Its Recycling for Growth and Its Pretreatment

Extracellular algal organic (EOM) matter released by the microalgae during its growth was analyzed for its total organic carbon (TOC), humic acid, and pigment contents. Analysis was conducted using a TOC analyzer ( TOC-V_CSN_, Shimadzu) and UV–vis spectrophotometer (UV-2600i, Shimadzu). The growth medium was recycled three times without any pretreatment. After the third cycle, water was treated with commercial granular activated carbon (Sigma Aldrich) to remove the EOM and pigments by measuring the absorbance at 254 and 440 nm, respectively (Mejia-da-Silva et al., 2018). All the macro- and micronutrients were added before each recycle.

## Results and Discussion

### Effect of Initial Nitrogen Concentration on Cell Growth and Metabolite Profile

Economical biofuel production and other valuable biochemicals from microalgae cannot be achieved without optimizing the supply of essential inputs such as nutrients, light, and CO_2_. The nutrients are expensive and have inherent greenhouse gas emissions (Li et al., 2019). Microalgae store excess nutrients, such as nitrate and phosphate, to utilize during their unavailability in the media (Bernard, 2011). This study investigates the impact of four initial nitrate concentrations as nitrogen (15.8, 31.6, 47.4, and 63.2 ppm) in modified BBM during the growth of microalgae *P. kessleri* HY-6 at a light intensity of 30, 60, and 100 μmol m^−2^ s^−1^. Supplementary Figures S1A,B show the cell growth at different nitrogen concentrations for two different light intensities. The data showed that additional nitrogen did not improve the biomass content (Zarrinmehr et al., 2020).

**FIGURE 1:**
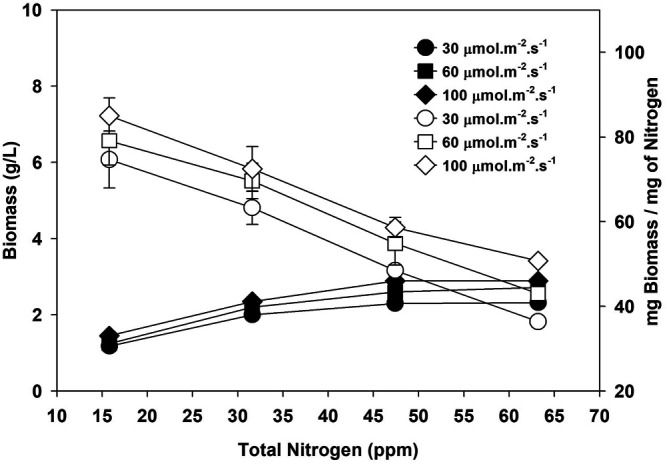
Biomass (g/L, filled symbols) and biomass per unit nitrogen (empty symbols) at three different light intensities.

In contrast, the specific growth rate reached a maximum of 0.36 day^−1^ at 47.4 ppm at 30 μmol m^−2^ s^−1^. Specific growth rate, doubling time, and biomass productivity was not affected much at higher nitrogen concentrations, and their values at two different nitrogen concentrations (47.4 and 63.2 ppm) are 0.3 day^−1^, 2.3 days, and 167 mg/L/day, respectively. The lipid content decreased from 46.7 to 26.5%, with an increase in nitrogen from 15.8 to 63.2 ppm at 60 μmol m^−2^ s^−1^, respectively. However, the microalgae attained the maximum lipid productivity of 56.2 mg L^−1^ day^−1^ at a nitrogen concentration of 47.4 ppm at 60 μmol m^−2^ s^−1^. These preliminary findings imply that the optimal compromise between maximizing the cell biomass and lipid productivity occurs at 47.4 ppm of nitrogen concentration under experimental conditions. Nutrient uptake depends on light intensity during growth, as shown in Supplementary Figures S2A,B. A higher light intensity (below light inhibition limit) increases photosynthesis, produces more biomass, and consumes more nitrogen (Khalili et al., 2015). Similar to this study, most studies used algal biomass and lipid productivity as the objective functions to maximize against nitrogen and other nutrients in the microalgae culture (Khalili et al., 2015; Zarrinmehr et al., 2020). However, optimizing biomass and CO_2_ fixation per unit amount of nitrogen will be a more realistic parameter. Maximizing biomass and CO_2_ fixation with the lowest input of nutrients is necessary to reduce the cost associated with nutrient supply (Selvaratnam et al., 2016). The data in Figure 1 shows that the total biomass (g/L) and the biomass per unit amount of nitrogen are functions of nitrogen concentration and depend on light intensity. Therefore, finding the light inhibition limit for the algae before doing any other optimization is recommended because light is the energy source to derive the photosynthesis process.

Figure 1 shows that biomass per unit nitrogen is higher at lower nitrogen and decreases as the nitrogen input increases. Total biomass increased with nitrogen, and based on biomass, the optimum nitrogen in the media should be around 47.4 ppm. However, the biomass productivity per unit amount of nitrogen is higher at low nitrogen content, *i*.*e*.,15.8 ppm for all three light intensities. Maximizing the biomass productivity at the lowest nitrogen consumption is desirable to decrease the cost of synthetic fertilizer and its associated greenhouse gas emissions. Total CO_2_ emission during synthetic fertilizer manufacturing varies according to the type of fertilizer. Ammonium nitrate and urea release 1.1–3.6 and 0.88–1.3 kg of CO_2_ equivalent per kilogram of product, respectively (Hoxha and Christensen, 2018). Nitrogen is the second most abundant element in algae biomass after carbon. So, an initial low nitrogen input will decrease fertilizer use and limit the unassimilated counter-ion concentration in media (Na^+^ in case of NaNO_3_), which is desired for water recycling for microalgae cultivation (Kumar and Bera, 2020). Nitrogen and phosphorus produced using fossil fuel energy required approximately 56.8 and 33.3 MJ/kg, respectively (Tredici et al., 2015). Biomass and energy content increased to 64, 112, and 125% when nitrogen was increased from 15.8 to 31.6, 47.4, and 63.2 ppm. The nitrogen concentration of 47.4 ppm seemed optimal as the net energy in biomass is 27.8, 45.5, 60.11, and 62.18 MJ/kg at 15.8, 31.6, 47.4, and 63.2 ppm of nitrogen, respectively, after accounting for the energy required for nitrogen production.

Moreover, pretreatment of algal bio-oil will be desired if biomass with high nitrogen is processed *via* hydrothermal liquefaction (Das et al., 2021; Farooq, 2021). Biomass per unit nitrogen is high at the lowest nitrogen content, but low total biomass per liter (in the case of 15.8 ppm nitrogen) will increase the harvesting cost. Harvesting is one of the expensive stages in the microalgae process, and culture density significantly affects the cost of harvesting. The cost of harvesting using centrifuge varies from 1.3 to 8 kWh/m^3^, depending on the culture concentration and harvesting efficiency. A low-density culture will consume more energy to recover a unit mass of microalgae (Dassey and Theegala, 2013). Though the biomass per unit nitrogen is high at low nitrogen (15.8 ppm), the overall biomass per liter of culture is low at this concentration, leading to higher harvesting costs. The energy required for harvesting biomass to produce 1 L of oil decreased with an increase in biomass per liter and its lipid content of biomass. The HHV of microalgae biomass is around 22 MJ/kg (Coimbra et al., 2019). The energy embedded in microalgae biomass produced at 15.8, 31.6, 47.4, and 63.2 ppm at 100 μmol m^−2^ s^−1^ is 9.2, 13.4 17.11, and 17.72 kWh/m^3^, respectively. Approximately 14% of the energy in biomass grown at 15.8 ppm will be used if the centrifugal harvesting option with minimum energy (∼ 1.3 kWh/m^3^) is explored. The energy utilized for harvesting biomass will further increase to 86% of total energy in biomass if harvesting needs 8 kWh/m^3^ of energy using centrifugation. So, a nitrogen concentration of 47.4 ppm was selected for the next experiment.

**FIGURE 2:**
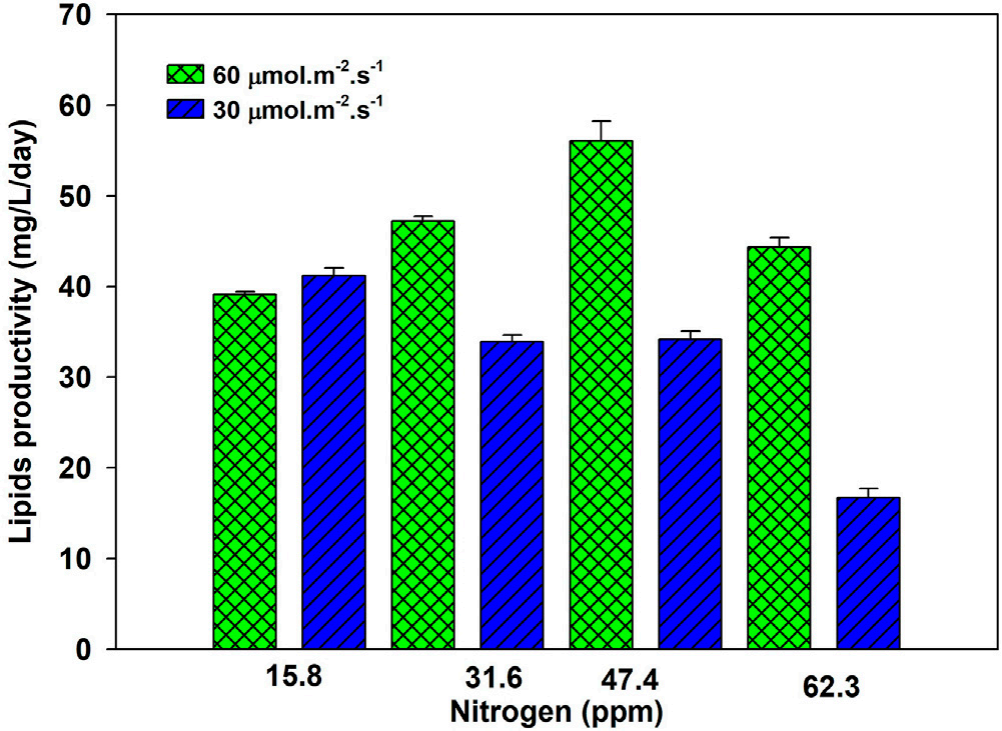
Lipid productivity of *Parachlorella kessleri* HY-6 at different nitrogen concentrations under 30 and 60 μmol m^−2^ s^−1^ under 2% CO_2_.

Lipid productivity first increased and then decreased at the same light intensity at varying nitrogen concentrations. A higher nitrogen concentration gives lower lipid productivity. Lipid productivity differed at two tested light intensities, as shown in Figure 2. This observation highlights the importance of supplying optimal light and nutrients. Lipid productivity is the product of biomass and lipid contents, an increase of both or any one will increase the lipid productivity (Sharma et al., 2012). An increase in lipid productivity with an increase in nitrogen concentration remains almost the same over the tested nitrogen concentrations. An increase in nitrogen from 15.8 to 31.6 ppm increased the lipid productivity from 39 to 48 mg/L/day. A further increase in nitrogen from 31.6 to 47.8 ppm increased the lipid productivity to 55 mg/L/day. These results showed that increasing the nitrogen beyond 47.4 ppm was not practical to enhance lipid productivity. Supplementary Figure S2 shows that 99% of nitrogen at all initial concentrations, except 63.4 ppm, was assimilated within 6 days. Light intensity drives the photosynthesis process, which promotes cell growth. At low light intensity, growth is low, and the rate of nitrogen update from media is low, as shown in Supplementary Figure S2.

### Effect of NP Ratio on the Cell Growth and Lipid Profile of *P. kessleri* HY-6

**FIGURE 3:**
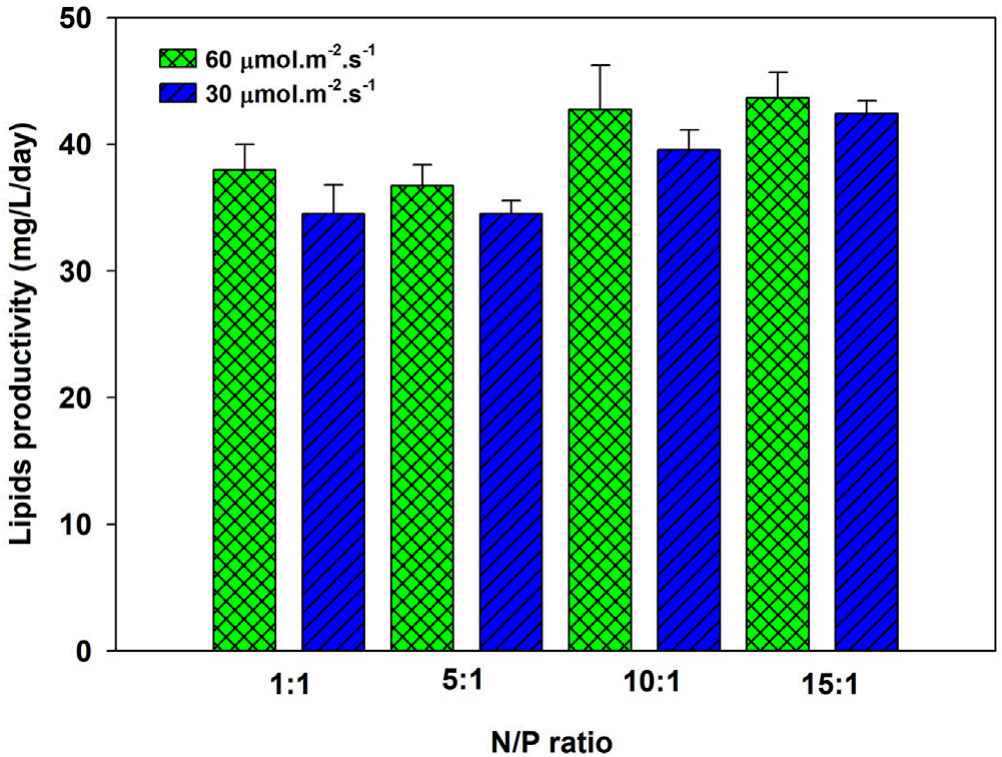
Lipid productivity of *Parachlorella kessleri* HY-6 at different N/P ratios at 60 μmol m^−2^ s^−1^ and 2.0% CO_2_.

The microalgae biomass contains approximately 1% of phosphorus, depending on the species. Due to limited reserves, optimizing phosphorus as macronutrients is essential without compromising biomass productivity. The nitrogen to phosphorus (N/P) ratio affects the cell growth and lipid accumulation of microalgae (Rasdi and Qin, 2015). *P. kessleri* HY-6 was cultivated in modified BBM media at four different TP concentrations (47.4, 9.5, 4.86, and 3.17 ppm) at 60 μmol m^−2^ s^−1^, which corresponds to NP ratios of 1:1, 5:1, 10:1, and 15:1 while keeping the nitrogen content at 47.4 ppm. The growth at different NP ratios is shown in Supplementary Figure S3, which is similar at different N/P ratios. The specific growth rate increased with the increase in the N/P ratio and reached the maximum at 0.45 day^−1^ at 10:1 NP ratio. Nitrogen is fully consumed at all N/P ratios. However, phosphorus uptake was slower at a lower N/P ratio, such as 1:1, as shown in Supplementary Figure S4. Overall, nitrogen and phosphorus were almost entirely consumed by microalgae at an NP ratio between 5:1 and 15:1. The nitrogen removal efficiency decreased due to phosphorus limitation (Xin et al., 2010). The total lipid of *P. kessleri* HY-6 under different NP ratios varies with the N/P ratio (Rasdi and Qin, 2015). The lipid productivity reached as high as 46.0 mg L^−1^ day^−1^ at 10:1 and 15:1 N/P ratios. Nutrient limitation has a significant effect on improving the lipid content of algal biomass (Rodolfi et al., 2009). Goldberg and Cohen stated that the total lipids, such as triacylglyceride, increased substantially from 6.5 to 39.3% under limited phosphorus (Khozin-Goldberg and Cohen, 2006). The lipid content of 45.0% was estimated during the study, leading to the lipid productivity rate of 46.0 mg/L/day at N/P ratio 10:1, under aeration with 2.0% CO_2_, as shown in Figure 3.

### Effect of Light and CO_2_ Concentration on the Cell Growth, Heating Value, and Biochemical Composition of *P. kessleri* HY-6

The microalgae *P. kessleri* HY-6 was cultivated at 25 ± 2°C at various light intensities (30, 60, and 100 μmol m^−2^ s^−1^) at different CO_2_ concentrations in a modified media with an N/P ratio of 10:1. Optimal light intensity and wavelength are desired for the growth of microalgae. Too low and too high light intensity negatively affect the microalgae growth (Jiang and Zheng, 2018). Earlier studies showed species-dependent growth inhibition at higher CO_2_ concentrations (Lim et al., 2021). The microalgae *P. kessleri* HY-6 can grow at a relatively high (6–15%) CO_2_ concentration, as shown in Supplementary Figure S5. Algal biomass increased with CO_2_ concentration at all three light intensities. However, the maximum biomass obtained at an optimal CO_2_ concentration varies with light intensity. At 2% CO_2_ and 30 μmol m^−2^ s^−1^, the maximum biomass was 1.66 g/L. The maximum biomass at 6.0 and 10.0% CO_2_ was 1.88 and 2.0 g/L at a light intensity of 60 and 100 μmol m^−2^ s^−1^. Both light and CO_2_ concentration enhanced the specific growth rate of microalgae, as shown in Supplementary Figure S6.

Supplementary Figure S7 shows the variation in the productivity of metabolites (lipid, carbohydrate, and protein) at different CO_2_ concentrations. The relative amounts of lipids, carbohydrate, protein, and pigment content of biomass are given in Table 1. The lipid content increased under the higher light intensity of 100 μmol m^−2^ s^−1^. At the same time, the pigment amount decreased, which could be due to stress conditions at a high light intensity and nitrogen-depleted conditions at the end of the growth stage (Liang et al., 2020). Variation in metabolites content is a complex function of various biotic and abiotic growth factors, such as light intensity, CO_2_ availability, pH, and nutrient level in the growth media (Gonçalves et al., 2014; Thawechai et al., 2016). The microalgae convert CO_2_ to biomass *via* a carbon capture mechanism in the presence of light. At high light intensity, growth will be fast, and nutrients will deplete quickly compared at low light intensity for the same cultivation time. Under the nutrient-replete condition, the protein and carbohydrate contents increased in biomass, while the lipid content increased during nutrient-depleted growth. However, this metabolite modulation is species specific as well. The lipid compositions at various CO_2_ concentrations and light intensity are provided in Supplementary Table S1. The data clearly showed that the total FAME content increased with CO_2_ and light intensity. The increasing light intensity increased the amount of saturated fatty acids, such as pentadecanoic acid (C15:0), palmitic acid (C16:0), and stearic acid (C18:0), with a significant variation in C16:0 and C18:0. However, the major portion of fatty acids are palmitic acid and oleic acid (C18:1) (Thawechai et al., 2016). The relative amount of unsaturated fatty acids (C18:1, C18:2, and C18:3) is higher than that of saturated fatty acids. However, the fatty acid composition variation depends on species, light intensity, and light exposure duration for cells (Nzayisenga et al., 2020).

**TABLE 1:** Biochemical composition of microalgae biomass under different light and CO_2_ conditions.

CO_2_ level	Light intensity (μmol/m^2^ s)	Biomass content (%)				
		Lipid	Protein	Carbohydrate^a^	Pigment	Ash
Air	30	37.9 ± 3.0	21.5 ± 0.6	33.4 ± 3.4	0.019 ± 0.005	7.2 ± 0.4
	60	24.9 ± 0.7	20.4 ± 0.1	47.2 ± 0.8	0.012 ± 0.002	7.6 ± 1.1
	100	32.0 ± 2.6	16.2 ± 0.4	50.0 ± 2.6	0.005 ± 0.003	1.7 ± 0.9
2%	30	41.5 ± 1.3	18.3 ± 0.7	32.1 ± 1.6	0.015 ± 0.006	8.1 ± 0.6
	60	26.9 ± 0.8	16.3 ± 0.1	51.6 ± 1.1	0.013 ± 0.006	5.2 ± 2.1
	100	45.1 ± 0.8	9.2 ± 0.1	40.4 ± 1.4	0.003 ± 0.001	5.3 ± 0.7
4%	30	32.4 ± 2.7	21.3 ± 0.2	45.6 ± 3.7	0.024 ± 0.002	0.6 ± 0.4
	60	33.0 ± 3.5	16.3 ± 0.4	49.6 ± 3.1	0.024 ± 0.004	1.1 ± 2.5
	100	47.6 ± 2.7	12.2 ± 0.4	39.4 ± 4.2	0.005 ± 0.001	0.9 ± 0.7
6%	30	29.5 ± 2.7	20.0 ± 0.7	49.8 ± 3.6	0.020 ± 0.001	0.6 ± 0.1
	60	34.0 ± 1.3	18.5 ± 0.4	46.0 ± 2.1	0.018 ± 0.003	1.5 ± 0.9
	100	47.7 ± 0.5	12.9 ± 0.6	37.5 ± 0.8	0.011 ± 0.005	1.9 ± 1.6
10%	30	25.9 ± 1.4	21.4 ± 1.8	52.0 ± 1.8	0.022 ± 0.003	0.8 ± 0.6
	60	37.8 ± 0.4	18.3 ± 0.0	43.3 ± 0.6	0.019 ± 0.001	0.6 ± 2.3
	100	59.9 ± 1.2	11.0 ± 0.6	28.3 ± 2.0	0.006 ± 0.004	0.7 ± 1.1
15%	30	32.9 ± 0.2	21.3 ± 1.4	45.2 ± 0.3	0.022 ± 0.007	0.5 ± 0.3
	60	33.9 ± 0.3	19.9 ± 1.7	45.7 ± 0.4	0.019 ± 0.002	0.5 ± 1.2
	100	55.7 ± 0.7	12.7 ± 0.1	29.6 ± 1.0	0.004 ± 0.005	1.9 ± 1.4

a Carbohydrate = 100 − lipids − protein − pigments − ash.

**FIGURE 4:**
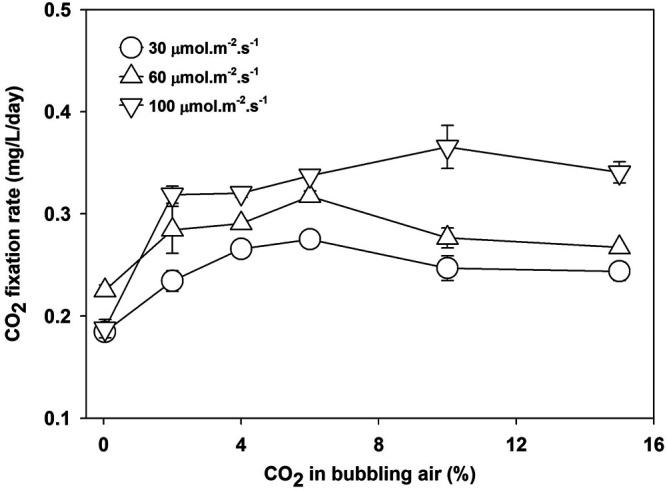
CO_2_ fixation rate of *Parachlorella kessleri* HY-6 at different light intensities and CO_2_ concentrations.

The CO_2_ fixation rate is calculated at a different light intensity and CO_2_ concentration using the carbon contents of biomass (given in Table 2) and Eq. 7. The CO_2_ fixation rate increased with the availability of CO_2_ in the media and the availability of light. However, the maximum CO_2_ fixation rate depends on light intensity. Under light-limited conditions, the photosynthesis process becomes less efficient, and the majority of CO_2_ will leave the system. The results in Figure 4 show that a CO_2_ concentration beyond 6% is not helpful as the fixation rate at 30 and 60 μmol m^−2^ s^−1^ decreased. However, the CO_2_ fixation rate increased at 10% CO_2_ concentration at 100 μmol m^−2^ s^−1^.

**TABLE 2:** Ultimate and proximate analysis of microalgae biomass obtained at different light intensities and CO_2_ concentrations.

CO_2_ level	Light intensity	Ultimate analysis			Proximate analysis			—	—	—
		C	H	N	HHV (MJ/kg)	MC	VM	FC	Ash	HHV (MJ/kg)
Air	30	42.7	6.5	3.6	15.5	9.0	71.0	12.9	7.2	16.8
	60	50.0	8.7	3.4	18.9	7.1	72.3	13.0	7.6	17.1
	100	48.7	8.6	2.7	18.4	7.1	72.3	18.9	1.7	18.5
2%	30	47.7	7.6	3.1	17.6	6.8	72.2	12.9	8.1	17.0
	60	52.1	9.7	2.7	20.0	2.6	73.8	18.5	5.2	18.7
	100	52.6	10.4	1.6	20.5	4.1	80.8	9.8	5.3	17.9
4%	30	52.6	9.2	3.4	19.9	6.9	75.4	17.1	0.6	18.7
	60	53.4	9.4	3.1	20.3	6.0	76.1	16.8	1.1	18.7
	100	55.2	12.0	2.2	22.2	3.7	82.7	12.7	0.9	19.0
6%	30	54.7	9.7	3.6	20.8	5.7	77.4	16.2	0.6	18.8
	60	54.1	9.7	2.7	20.6	5.1	77.9	15.5	1.5	18.8
	100	57.2	11.4	2.0	22.4	3.9	82.6	11.5	1.9	18.6
10%	30	53.5	7.3	3.6	19.2	5.7	77.1	16.4	0.8	18.8
	60	55.0	9.4	3.1	20.7	5.9	76.8	16.8	0.6	18.9
	100	60.3	11.2	1.9	23.3	2.7	86.0	10.6	0.7	19.0
15%	30	53.6	9.4	3.6	20.3	6.3	75.0	18.1	0.5	18.9
	60	55.5	9.2	3.4	20.8	5.5	79.6	14.4	0.5	18.8
	100	61.3	10.8	2.1	23.4	3.0	83.7	11.4	1.9	18.8

**FIGURE 5:**
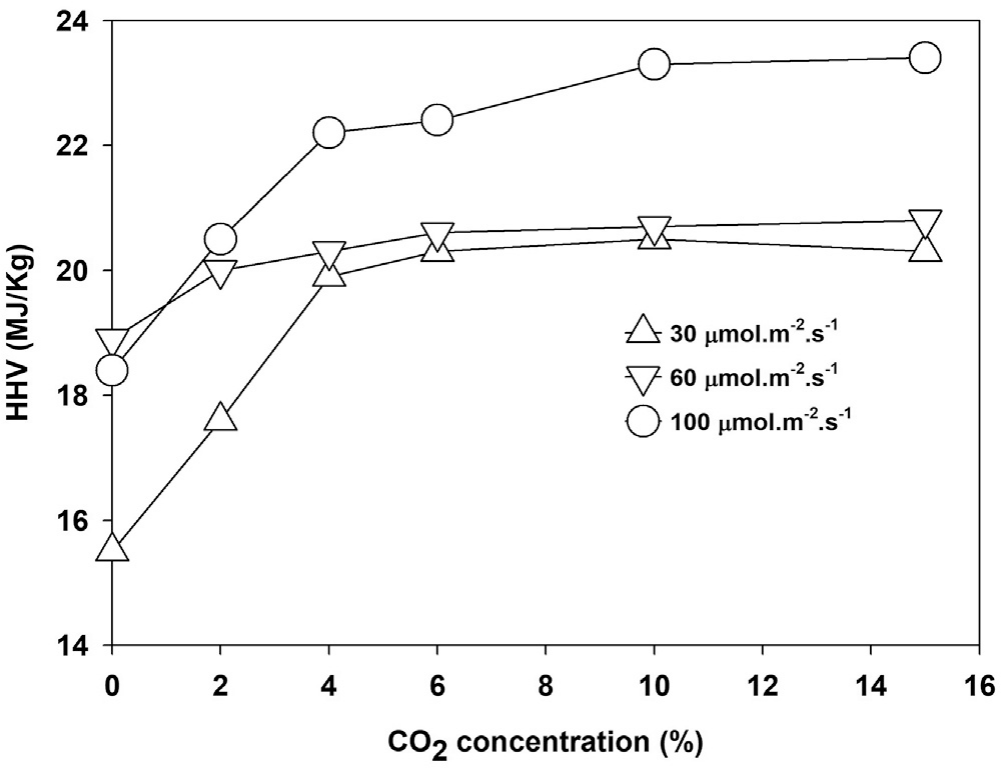
The higher heating value of *Parachlorella kessleri* HY-6 at different light intensities and CO_2_ concentrations.

The energy content of biomass as HHV increased with light intensity and CO_2_ concentration. The maximum HHV of 20, 20.5, and 22.50 MJ/kg was obtained at 6.0% CO_2_ for 30 and 60 μmol m^−2^ s^−1^ and at 10% for 100 μmol m^−2^ s^−1^, respectively, as shown in Figure 5. Growth under higher light intensity and CO_2_ concentration increased the CO_2_ fixation rate and enhanced the HHV value of the biomass, as given in Table 2.

**FIGURE 6:**
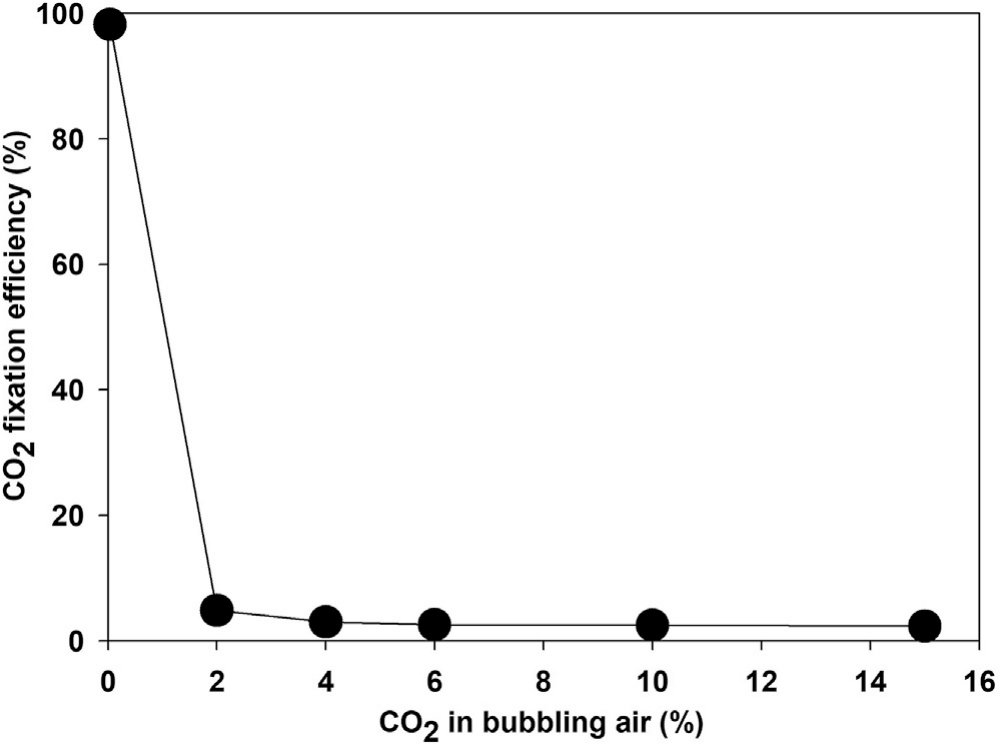
CO_2_ fixing efficiency by microalgae *Parachlorella kessleri* HY-6 at a different CO_2_ concentration.

A higher CO_2_ concentration enhanced the growth rate of microalgae compared to atmospheric air—for example, the growth rate increased from 0.301 to 0.527 day^−1^ when bubbling of atmospheric air switched with air containing 15% CO_2_. Though the biomass yield, CO_2_ consumption, and energy content of the microalgae increased at higher concentrations of CO_2_, the CO_2_ fixation efficiency declined sharply with an increase in CO_2_ concentration in the bubbling air (Lim et al., 2021). The CO_2_ removal efficiency was 96% when atmospheric air was supplied as a source of CO_2_. The removal efficiency decreased sharply to 2.5, 1.2, and 0.85% at a CO_2_ concentration of 2, 4, and 6%, respectively, as shown in Figure 6. The CO_2_ removal efficiency depends on microalgae strain, culture pH, bubble size, and hydraulic retention time in the photobioreactor, along with effective light availability (Thawechai et al., 2016). Most studies reported CO_2_ fixation by using the carbon content of microalgae rather than the direct measurement of CO_2_ at the outlet, which ignores the release of fixed carbon as EOM and dissolved CO_2_ in the water (Leflay et al., 2021). Estimation of EOM during the algae growth as an organic form of CO_2_ is necessary for finding the true potential of CO_2_ bio-fixation.

Most CO_2_ is released back to the atmosphere under low fixation efficiency (Lam and Lee, 2013). These results showed that the potential of microalgae to fix CO_2_ is debatable. Low CO_2_ fixation efficiency would be counterproductive at high CO_2_ concentrations if the energy was spent on acquiring CO_2_ from emission sources such as power plants and industrial flue gases. CO_2_ capture cost varies between 292 and 425 kJ/kg. Low CO_2_ fixation efficiency means loss of almost. One of the main reasons for low bio-fixation efficiency is the lower solubility of CO_2_ in water (∼ 1.45 g/L) at normal conditions (Daneshvar et al., 2022). The CO_2_ solubility and uptake efficiency can be enhanced by controlling the bubble size, design of the photobioreactor, proper selection of microalgae strain, and selecting appropriate operating conditions, such as flow rate, the concentration of CO_2_, and pH (Li et al., 2013). Therefore, future research should focus on improving the CO_2_ fixation efficiency at higher CO_2_ concentrations.

### Reuse of Water During Cultivation of Microalgae *P. kessleri* HY-6

Water reuse during microalgae cultivation is essential to reduce the high-water footprint of the cultivation stage (Farooq et al., 2015b; Farooq, 2021; Lu et al., 2020). Many researchers investigated the potential benefits and challenges associated with water reuse during microalgae cultivation. The studies on water recycling are essential for various reasons, such as cost of water itself, cost of water acquiring, loss of nutrients, pretreatment of water if not recycled, and presence of growth-promoting and growth-inhibiting organics released during the former stage (Chen L. et al., 2017; Mejia-da-Silva et al., 2018).

**FIGURE 7:**
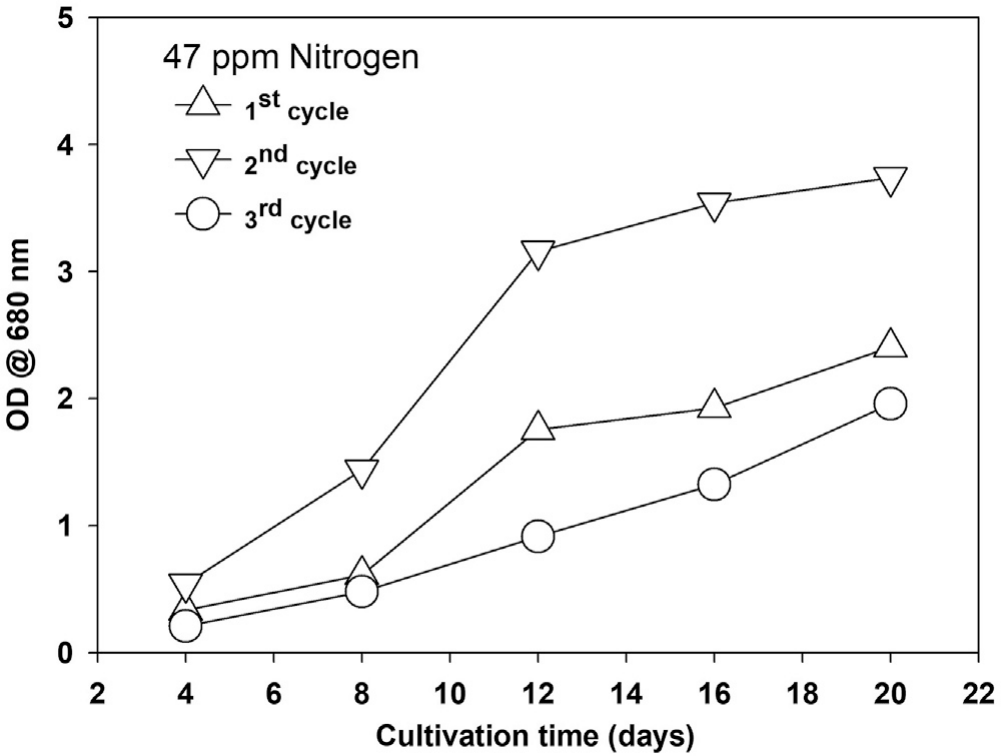
Growth of *Parachlorella kessleri* HY-6 in recycled water at 47 mg/L of nitrogen.

Microalgae growth was enhanced during the first recycle and then decreased in the second recycle, as shown in Figure 7 and Supplementary Figure S8A for two different nitrogen concentrations at 2% CO_2_.

**FIGURE 8:**
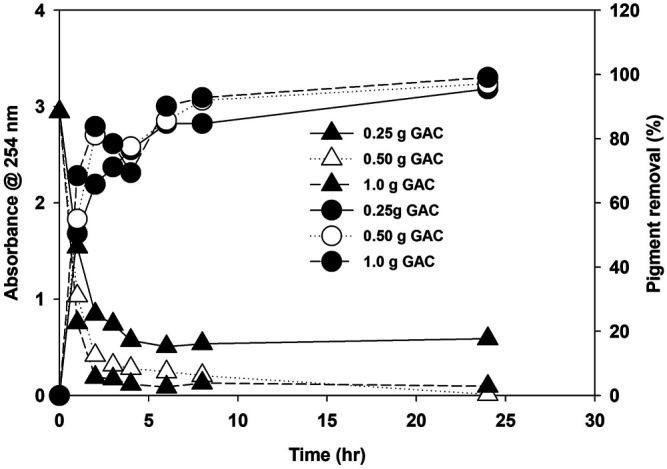
Removal of organic carbon (*A*_254 nm_) and pigments (*A*_440 nm_) at different amounts of granular activated carbon.

The possible reason for growth enhancement during the second recycle could be the growth-promoting organic released by microalgae in the first growth cycle. EOM released during growth are reported for their species-specific growth-promoting and growth-inhibiting role during microalgae cultivation (Liu et al., 2016). Microalgae release various types of extracellular organics during their growth, and their composition and amount depend on the growth conditions and are affected by many biotic and abiotic factors (Loftus and Johnson, 2019; Villacorte et al., 2015). The nature of EOM varies with the growth stage and is also affected by nitrogen availability in the media. Under nitrogen-replete conditions, most EOM is composed of protein, while under nitrogen-deplete conditions, adopted for lipid induction, EOM is mainly composed of polysaccharides (Baroni et al., 2020). Growth was severely inhibited during the third cycle, and bacteria were observed. Extracellular organics act as a source of bacterial growth. The microalgae growth rate was less during the third stage and showed the stress conditions at the early growth stage. The presence of unused salt ions accumulated during water recycling and increased organic load could be the potential growth-inhibiting factors (Sha et al., 2019). TOC was measured as the indicator of the accumulation of organic matter. TOC increased with each cycle at three different nitrogen concentrations, as shown in Supplementary Figures S8B. A decrease in TOC in the fourth cycle was observed, and during this stage, severe bacterial contamination was noticed, and the color of the media turned yellow. Various organics, including humic acids, protein, carbohydrates, and free fatty acids, contribute to TOC (Lu et al., 2019; Sha et al., 2019). Water after the third cycle was treated with a different amount of commercially available granular activated carbon (GAC) to remove the organics and pigment from the recycled water by measuring the absorbance at 254 and 440 nm, respectively. The GAC effectively removed the organic matter composed of various organics and pigments from the culture, as shown in Figure 8. Activated carbon is found effective for polishing the recycled water as reported, and a similar growth was observed as in the first cycle (Mejia-da-Silva et al., 2018), but the use of GAC will add to another cost factor as, for >95% removal, 1.0 g of GAC is required for 50 ml of water besides the cost of mixing in our study. Though regeneration of GAC is an option, the amount of GAC will be pretty significant considering the amount of water required to produce 1 L of biodiesel from microalgae biomass (Rocha et al., 2015). This finding suggests that limiting the release of organics and accumulation of unused ions are the first steps toward water recycling in microalgae besides the effective and economical treatment of recycled water. Moreover, the presence of a higher amount of EOM limits the performance of the harvesting system, especially membrane-based system (Zhang et al., 2018).

### Limitations and Recommendations for Future Work

This study investigates the impact of initial nitrogen and nitrogen-to-phosphorus ratio on microalgae growth, CO_2_ fixation efficiency, and water recycling during cultivation. Despite this initial investigation and valuable results, this study has not considered the impact of optimal light intensity based on the light saturation intensity of the algae under investigation. Light is a source of energy for photosynthesis, and finding the light saturation limit of microalgae is important before optimizing other growth paraments like nutrients and CO_2_ supply. Therefore, firstly, the value of light saturation is essential. Secondly, CO_2_ fixation efficiency must be tested under an optimal bubble size and flow rate according to culture depth. Thirdly, activated carbon was tested to remove the organic compounds as total organic carbon. Some organic molecules like carbohydrates and peptides support microalgae growth compared to secreted free-fatty acids and humic substances. Therefore, further studies should focus on the removal rate of different organics in the reused water. Adsorbents like activated carbon can remove the nutrients essential for growth as well. In this study, nutrients like nitrogen and phosphorus were consumed entirely by the microalgae. However, the role of activated carbon for removing unutilized cations (Na^+^, K^+^, Ca^+2^, and Mg^+2^) and anions (NO_3_^−1^, PO_4_^−3^, SO_4_^−2^, *etc*.) must be explored. Loss of CO_2_ as secreted organic matter during growth must also be accounted for toward the bio-fixation of CO_2_ by the microalgae.

## Conclusion

The growth and nutrient optimization for *P. kessleri HY-6* are investigated as algal biomass, lipid productivity, CO_2_ fixation efficiency, and water recycling potential under different nutrients and CO_2_ concentrations during photoautotrophic cultivation mode. The optimum total nitrogen concentration is 47.4 ppm at fixed environmental conditions of CO_2_ and light intensity. *P. kessleri* HY-6 efficiently utilized the nutrients and produced higher lipid productivity at an N/P ratio of 10:1. The CO_2_ fixation rate increased with an increase in CO_2_ concentration in aerated air, but the efficiency of CO_2_ fixation decreased drastically at 2% CO_2_ in the air. The CO_2_ fixation rate increased with light intensity from 30 to 100 μmol m^−2^ s^−1^. The HHV increased from 15.5 to 21 MJ/kg when the CO_2_ concentration was increased from 0.04% (ambient air) to 10% CO_2_. HHV also increased from 15.5 to 23.5 MJ/kg when the light intensity was increased from 30 to 100 μmol m^−2^ s^−1^. Water reuse was effective in improving the microalgae growth until second recycling. Further reuse inhibited the algae and enhanced the bacterial growth, resulting in poor CO_2_ utilization and nutrient uptake. Granular activated carbon (1.0 g/L) was effective for removing 95% of organic matter and pigments in 6 h, which is desired for growth in recycled water.

## Data Availability

The original contributions presented in the study are included in the article/Supplementary Material, further inquiries can be directed to the corresponding author.

## Supplementary Material

Supplementary Table S1 FAME composition of *Parachlorella kessleri* HY-6 under different CO_2_ levels and light intensities.
