# Supplementary material for: Maximizing Energy Content and CO2 Bio-fixation Efficiency of an Indigenous Isolated Microalga Parachlorella kessleri HY-6 Through Nutrient Optimization and Water Recycling During Cultivation
Source: Front Bioeng Biotechnol. 2022 Feb 10;9:804608. doi: 10.3389/fbioe.2021.804608 (PMC8867024; doi:10.3389/fbioe.2021.804608)
Supplement: Supplementary file 1 [file DataSheet1.docx]

Supplementary information

Figure 1s. Growth of *Parachlorella kessleri* HY-6 at (a) 30 μmol.m^-2^.s^-1^ and (b) 60 μmol.m^-2^.s^-1^ under 2.0 % CO_2_.

**(a)**

Figure 2s. Nitrogen removal and uptake by *Parachlorella kessleri* HY-6 at (a) 30 μmol.m^-2^.s^-1^ and (b) 60 μmol.m^-2^.s^-1^ under 2.0 % CO_2_

**
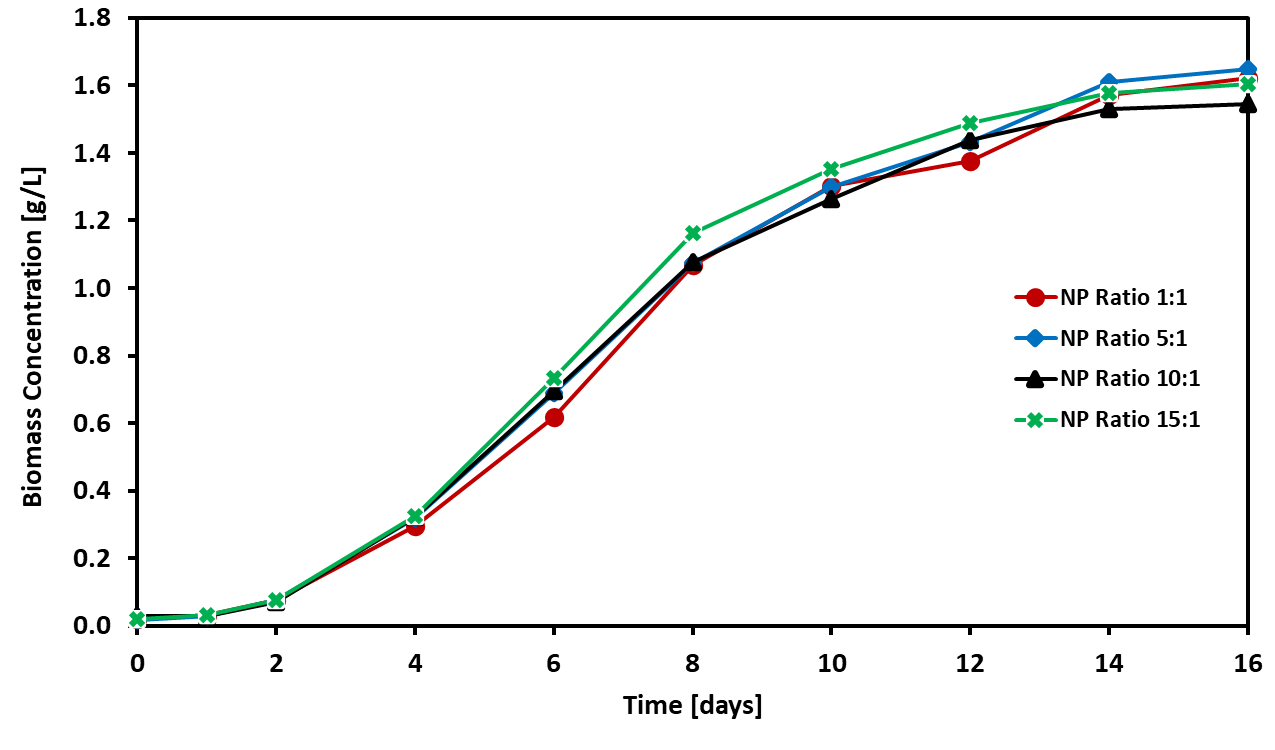
**

Figure 3s. Growth of microalgae *Parachlorella kessleri* HY-6 at different N/P ratios at 60 μmol.m^-2^.s^-1^ and 2.0 % CO_2_.

Figure 4s. Nutrient’s consumption at different N/P ratios during growth of microalgae *Parachlorella kessleri* HY-6 at different N/P ratios at 60 μmol.m^-2^.s^-1^ and 2.0 % CO_2_.

Figure 5s. Biomass of microalgae of *Parachlorella kessleri* at various light intensity and CO_2_ concentrations (a = 30 μmol.m^-2^.s^-1^, b = 60 μmol.m^-2^.s^-1^, c =100 μmol.m^-2^.s^-1^)


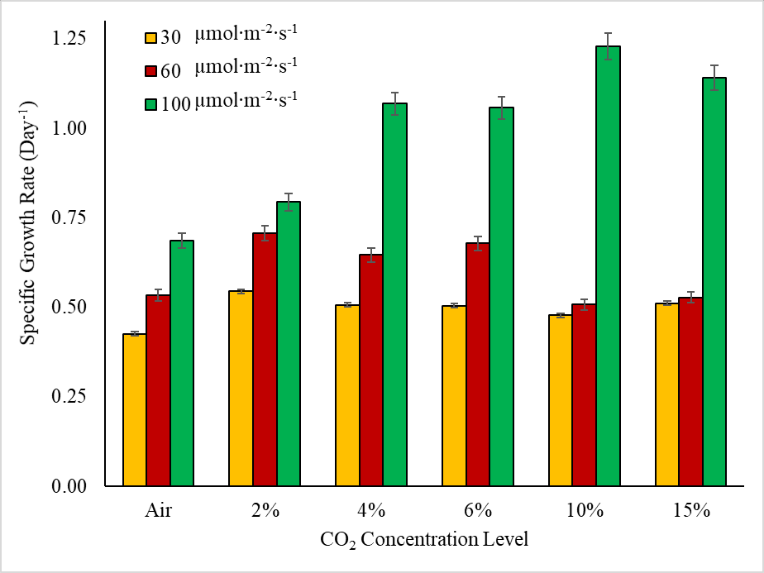


Figure 6s. The specific growth rate of microalgae *Parachlorella kessleri* HY-6 at different light intensity and CO_2_ concentrations.

Figure 7s. Variation in lipid, carbohydrate, and protein productivity of Parachlorella kessleri at a various light intensity and CO_2_ concentrations

(a)

(b)

Figure 8s. Growth of microalgae of *Parachlorella kessleri* during water recycling at 60 μmol.m^-2^.s^-1^ at (a) 32 ppm and (b) increase in total organic carbon (TOC) during water recycling
